# Usefulness of preprocedural 3-dimensional computed tomography planning in assisting one-stage pulmonary veins isolation with concomitant left atrial appendage occlusion procedure: A pilot study^☆^

**DOI:** 10.1016/j.ijcha.2024.101594

**Published:** 2024-12-30

**Authors:** Ke-Wei Chen, Yen-Nien Lin, Mei-Yao Wu, Yi-Hsiu Wu, Wen-Sheng Feng, Ping-Han Lo, Wei-Hsin Chung, Cheng-Chang Tung, Kuan-Cheng Chang

**Affiliations:** a Division of Cardiovascular Medicine, Department of Medicine, China Medical University Hospital, China Medical University, Taichung 404327, Taiwan; b Graduate Institute of Biomedical Sciences, China Medical University, Taichung 404333, Taiwan; c School of Medicine, China Medical University, Taichung 404333, Taiwan; d School of Post-Baccalaureate Chinese Medicine, China Medical University, Taichung 404333, Taiwan; e Department of Chinese Medicine, China Medical University Hospital, Taichung 404327, Taiwan; f Department of Mechanical Engineering, National Cheng Kung University, Tainan 701401, Taiwan; g AI Center for Medical Diagnosis, China Medical University Hospital, Taichung 404327, Taiwan; h Digital Transformation Technology Office, China Medical University Hospital, Taichung 404327, Taiwan

**Keywords:** Atrial fibrillation, Pulmonary vein isolation, Left atrial appendage occlusion, 3D computed tomography

## Abstract

**Background:**

The optimal imaging modality for selecting the device size in patients with atrial fibrillation undergoing one-stop left atrial appendage occlusion (LAAO) with concomitant pulmonary vein isolation (PVi) remains undefined. We compared preprocedural 3-dimensional computed tomography (3D CT) with intra-procedural transesophageal echocardiography (TEE) and left atrial appendage (LAA) angiography in guiding one-stage PVi and LAAO.

**Methods:**

We measured the LAA ostium diameter using an interactive 3D CT system with a central line-based approach and compared these measurements with those from intra-procedural TEE and angiography, and the actual device size. The optimal compression ratio was used to assess the attainment rates of the three imaging modalities.

**Results:**

Twenty-two patients (median age: 68.5 years, 21.8 % female) underwent the one-stage procedure. The median LAA ostium diameter measured by 3D CT (24.3 mm, interquartile range [IQR] = 22.0–27.0 mm) was closer to the Watchman device size (27.0 mm, IQR = 24.0–31.0 mm, P = 0.127) compared to TEE (21.2 mm, IQR = 18.4–22.7 mm, P < 0.001) and angiography (22.5 mm, IQR = 17.9–25.1 mm, P < 0.001). 3D CT had a better attainment rate for the optimal compression ratio than TEE (10.8 %, IQR = 7.4–16.5 % vs. 22.7 %, IQR = 19.2–29.3 %, P < 0.001) and angiography (19.7 %, IQR = 15.1–24.1 %, P = 0.001). All patients underwent successful device implantation without peri-device leak or complications during the periprocedural period and follow-up.

**Conclusions:**

In this pilot study, a preprocedural central line-based 3D CT planning system appeared to be more effective than intraoperative TEE and angiography in measuring the LAA ostium diameter to guide device size selection in patients with atrial fibrillation undergoing one-stop LAAO with concomitant PVi.

## Graphical abstract

**Figure:**
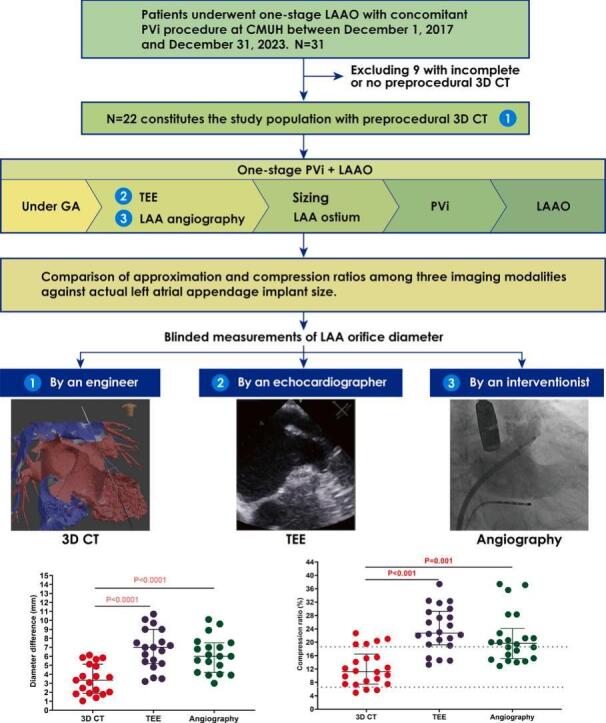

## 1 Introduction

The integration of pulmonary vein isolation (PVi) and left atrial appendage occlusion (LAAO) into a one-stage procedure is increasingly recognized for its benefits in relieving symptoms and reducing the risk of stroke in patients with atrial fibrillation (AF) [1], [2], [3], [4]. Typically, catheter-based ablation is performed before LAAO to minimize the risk of occluder malpositioning or dislodgement. However, acute tissue edema because of radiofrequency or cryoballoon ablation can alter the shape and size of the left atrial appendage (LAA) ostium [2], complicating the selection of an appropriate LAAO device size. This alteration can impact procedural success and increase the risk of peri-device leakage once the edema subsides. Strategies to address this issue include using a larger device to enhance the compression ratio despite the increased risk of LAA rupture, and performing LAA angiography before ablation. However, no consensus exists on the optimal LAAO device size for a one-stage procedure [1], [5].

Transesophageal echocardiography (TEE) is essential for guiding LAAO procedures and preprocedural planning, particularly for measuring the maximum diameter of the LAA ostium to determine the occlusion device size. However, radiofrequency or cryoballoon ablation can alter the LAA ostium shape because of edema, complicating intraoperative TEE assessment of the ostium size. This effect is more pronounced with cryoballoon ablation, which, owing to its larger endocardial contact area compared to radiofrequency ablation, may cause more extensive tissue damage and edema. The cryoballoon can also cause swelling of the pulmonary vein-LAA ridge, potentially affecting occluder stability [2], [6]. Therefore, the ability of TEE to accurately assess the LAA orifice area for optimal device selection in one-stage AF ablation with a concomitant LAAO procedure remains uncertain. Intracardiac echocardiography (ICE) has recently gained recognition as a valid alternative to TEE for intra-procedural guidance [7], [8]. ICE can be used to corroborate measurements obtained from pre-acquired computed tomography (CT) scans to size the LAA during the procedure.

In this study, we employed an innovative preprocedural three-dimensional (3D) CT planning approach for one-stage PVi with concomitant LAAO in patients with AF. We used an interactive central line-based 3D CT planning system to automatically determine the location of the LAA ostium and select the appropriate implant size within a 3D CT model. We aimed to compare the effectiveness of preprocedural 3D CT in assessing the LAA ostium diameter with intra-procedural TEE imaging and LAA angiography in guiding one-stop PVi with a concomitant LAAO procedure.

## 2 Methods

### 2.1 Study subjects

Thirty-one consecutive patients with AF who underwent a one-stage procedure involving the PVi and LAAO were enrolled in the study between December 1, 2017, and December 31, 2023. Nine patients were excluded because they had incomplete or no preprocedural 3D CT. Twenty-two patients were enrolled, and preprocedural 3D CT images were analyzed to assess the diameter of the LAA ostium.

### 2.2 Image processing

The patient underwent a 3D CT scan 1–4 weeks before the procedure. CT scans were analyzed preoperatively using a central line-based method by an engineer who was blinded to the clinical procedure. We saved the patients' CT images in the DICOM format, typically comprising 500–750 images per chest CT series. Using the Pydicom package, we parsed the DICOM files provided by the doctors’ post-CT scans. Our image processing workflow involved several steps, including filtering out non-essential images such as scout, non-CT, dose record, dose report, and screen-save images. We identified the main image series based on the “SeriesDescription” tag and the number of images in each series. To reconstruct the 3D images accurately, we used the “ImageOrientationPatient” tag to determine the X- and Y-coordinates (u- and v-directions) in the CT coordinate system. The Z-coordinate was derived from the outer product of the u and v vectors, with the “ImagePositionPatient” tag providing the image position along the Z-coordinate. Sequentially stacking the images along the Z-coordinate direction enabled the correct reconstruction of the patient's 3D image.

### 2.3 Interactive central line-based 3D CT planning

Similar to other medical imaging software, we converted the raw image data into Hounsfield units (HU), where soft tissues typically ranged below +60 HU, muscles were approximately +35 to +55 HU, and bones were between +300 and +1900 HU. Owing to the contrast agents, the blood vessels exhibited higher HU values than the soft tissues and muscles. Therefore, our algorithm sets a threshold above 60 HU to segment the blood vessel area, considering the CT imaging noise with a threshold typically between 100 and 200 HU. We employed a region-growing method to segment and label HU values surpassing the threshold, adjusting the threshold based on vessel CT intensity. To exclude bones, such as the spine, ribs, and sternum, that exceeded the threshold, we limited the growth range of the region-growing algorithm. Finally, we utilized the dual-marching cube algorithm to extract the isosurface of a 3D image and create a 3D mesh model.

The threshold value in the region-growing method is important in the modeling process. As most chest CT images are acquired horizontally, identifying the LAA opening position in this orientation can be challenging. To address this, we employed multiplanar reformation technology to reconstruct sagittal and coronal views. Users can freely switch between the horizontal, sagittal, and coronal views to observe the LAA opening and determine the threshold of the segmentation algorithm. To expedite the subsequent analyses, we utilized the edge collapse algorithm to simplify the mesh complexity of the heart model to one-fifth of its original form [9].

**Fig. 1:**
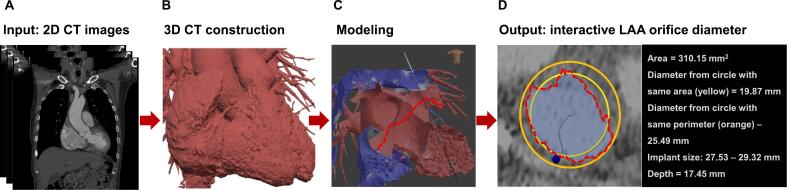
**Interactive Preprocedural 3D Computed Tomography (CT) Planning.** Using CT data for 3D modeling, we employed an interactive central line algorithm to analyze the central axis from the fossa ovalis puncture point to the tip of the left atrial appendage (LAA), automatically capturing the anatomical data of the LAA ostium. In the 'output' diagram, the red dashed line indicates the actual shape of the LAA ostium, the light-yellow circle represents a hypothetical circle with the same area as the LAA ostium, and the orange circle represents a hypothetical circle with the same circumference as the LAA. Based on this analysis, the area-derived diameter, perimeter-derived diameter, depth, and recommended implant size were determined for the preprocedural planning. (For interpretation of the references to colour in this figure legend, the reader is referred to the web version of this article.)

Analysis of the LAA opening involves three key components (Fig. 1). Initially, it was necessary to ascertain the precise location of the LAA opening and determine the angle of implant placement. Subsequently, the outline of the LAA opening, specifically, the region in which the implant was positioned, was calculated. Finally, the LAA perimeter, outline area, and depth were computed as part of the analysis.

To expedite the determination of the LAAO device position and angle, we used the Vascular Modeling Toolkit (VMTK, https://www.vmtk.org) to compute the centerline within the heart model. This centerline represents the path traversed by the cardiac catheter from the right atrium through the fossa ovalis to the end of the LAA. By setting the position of the LAAO device along this path, users can automatically ensure that its alignment is parallel to the centerline direction. However, manual configuration of the fossa ovalis and LAA end positions is necessary. We adopted the reciprocal of the maximum inscribed ball radius as the cost function for the centerline calculation.

After the user selects the position of the occluder along the centerline, a plane perpendicular to the centerline is created. The orientation of this plane approximates the opening direction of the LAA. If the angle of the plane does not meet the clinical requirements, the intersection point of the plane and the central path can be stabilized, enabling users to adjust the plane's angle precisely. Any plane position or angle modification triggered immediate automatic updates of the calculated data within the software.

In the occluder size analysis, calculations were performed to determine the intersection of a plane with the heart model, yielding multiple contour lines. To evaluate the contour of the LAA opening, we computed the winding number of the contour line around the centerline. Subsequently, the perimeters and areas of the identified contour lines were quantified. The engineer analyzing the interactive central line-based 3D CT planning system was blinded to the clinical procedures.

### 2.4 Intra-procedural TEE imaging and LAA angiography

An experienced echocardiographer performed the intra-procedural TEE. Measurements of the LAA ostium, including those obtained from conventional views at 0°, 45°, 90°, and 135°, as well as 3D TEE reconstruction, were performed prior to the ablation procedure. This approach was used to avoid anatomical alterations that may occur post-ablation, such as shifts in the entry axis or ostium deformation caused by edema of the pulmonary vein-LAA ridge. Simultaneously, LAA angiography was performed using a pigtail catheter in the RAO caudal view, and an experienced interventionist measured the maximum diameter of the LAA ostium for comparison. The echocardiographer and interventionist were blinded to the preprocedural central line-based 3D CT planning results. Based on the intraoperative TEE and LAA angiography measurements, the interventionists selected the appropriate size of the Watchman or Watchman FLX device according to their experience to achieve the desired compression rate that would ensure device stability. The device was deployed after confirming that it met the position, anchor, size, and seal criteria with TEE, thus completing the procedure.

### 2.5 Clinical follow-up

Qualified cardiologists reviewed the medical records to identify and adjudicate clinical outcomes. The optimal compression rate was defined as 8 %–20 % in the Watchman device and 10 %–30 % in the Watchman FLX device. All patients received post-procedural TEE to evaluate residual leakage, thrombosis, and device stability.

### 2.6 Statistical analysis

Continuous variables are expressed as mean ± standard deviation (SD) or median with interquartile range (IQR). We employed Kruskal–Wallis analysis to compare the LAA ostium diameter determined by interactive central line-based 3D CT planning, TEE, and intra-procedural LA angiography with the actual device size. Categorical data are presented as n (%) and were assessed using the chi-squared test. Statistical significance was defined as a two-sided P-value of < 0.05. All statistical analyses were performed using the SPSS software (version 12.0; SPSS Inc., Chicago, IL, US).

## 3 Results

We analyzed a cohort of 22 consecutive patients with AF, 78.26 % male, with a median age of 68.5 years (IQR = 59.3–74.5 years). These patients underwent one-stage PVi with concomitant LAAO (Table 1) because of ineffective or intolerant anticoagulation therapy. Among these patients, 36.36 % experienced cerebrovascular accidents, and hypertension and diabetes mellitus were prevalent in 68.18 % and 36.36 % of the cohort, respectively. The AF types were categorized as paroxysmal (68.18 %), persistent (27.27 %), and permanent (4.55 %). The average CHA_2_DS_2_-VASc score was 3.0 (2.0–4.0), with a HAS-BLED score of 2.0 (2.0–3.0). Preprocedural medications included antiplatelets (18.18 %), non-vitamin K antagonist oral anticoagulants (59.09 %), warfarin (13.64 %), and no medication (9.09 %). The left atrial volume index was 43.1 cm^3^/m^2^ (35.7–59.0 cm^3^/m^2^), and the left ventricular ejection fraction was 53.7 % (50.4 %–62.2 %).

**Table 1:** Demographics and clinical characteristics.

**Variables**	**N = 22**
**Age**	68.5 (59.3–74.5)
**Sex (male)**	17 (78.26 %)
**Comorbidities**	
Old cerebrovascular accident	8 (36.36 %)
Hypertension	15 (68.18 %)
Diabetes mellitus	8 (36.36 %)
Coronary artery disease	10 (45.45 %)
Chronic kidney disease (eGFR < 60 mL/min/1.73^2^)	9 (40.91 %)
**Type of atrial fibrillation**	
Paroxysmal	15 (68.18 %)
Persistent	6 (27.27 %)
Permanent	1 (4.55 %)
**CHA_2_DS_2_-VASC score**	3.0 (2.0–4.0)
**HAS-BLED score**	2.0 (2.0–3.0)
**Pre-procedure medication**	
Antiplatelet agents	4 (18.18 %)
Non-vitamin K antagonist oral anticoagulants	13 (59.09 %)
Warfarin	3 (13.64 %)
None	2 (9.09 %)
**Echocardiography**	
Left atrial volume index (cm^3^/m^2^)	43.1 (35.7–59.0)
Left ventricular ejection fraction (%)	53.7 (50.4–62.2)
**Indications of procedure**	
Intolerance of oral anticoagulants	16 (72.73 %)
Embolic event despite medication	6 (27.27 %)

Data are presented as median (IQR, interquartile range) and n (%).

**Fig. 2:**
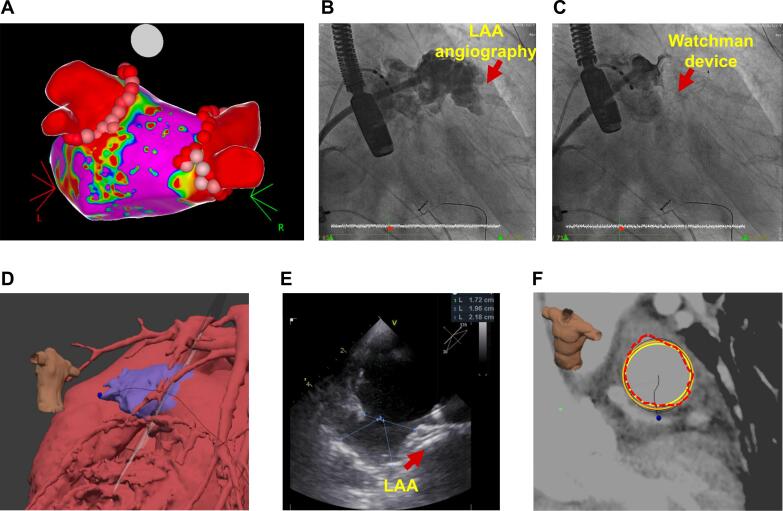
**Representative case of radiofrequency ablation with concomitant LAAO** In this representative case, we first performed an atrial septal puncture, followed by pulmonary vein isolation (PVi) using 3D mapping-guided radiofrequency ablation (A). This is followed by left atrial appendage (LAA) angiography (B) and implantation of an LAA occluder (C). Preprocedural CT planning images display various angles and automatically highlight the LAA (D). For comparison, intra-procedural transesophageal echocardiography (TEE) was performed (E). Analysis of the LAA ostium automatically calculated key anatomical data and provided recommendations for a suitable implant size (F).

**Fig. 3:**
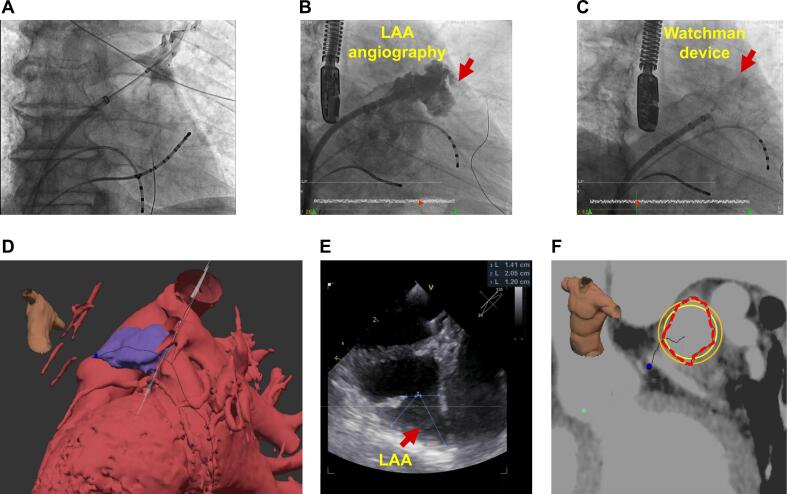
**Representative case of cryoballoon ablation with concomitant LAAO** In this representative case, we first performed an atrial septal puncture and pulmonary vein isolation (PVi) using a cryoballoon (A), followed by LAA angiography (B), and then implanted a left atrial appendage occluder (C). Preprocedural CT planning images display various angles and automatically highlight the LAA (D). For comparison, an intra-procedural transesophageal echocardiography (TEE) is shown in (E). Analysis of the LAA ostium automatically calculates key anatomical data and provides recommendations for suitable implant size (F).

Most PVi procedures (81.82 %) were conducted using radiofrequency (RF) catheter ablation (Fig. 2), whereas cryoablation was employed in 18.18 % of cases (Fig. 3). For LAAO, the Watchman device was used in 82.61 % of the cases, and the Watchman FLX device was used in 17.39 %. The one-stage procedural time, LAAO implant time, and total fluoroscopy time were 165.59 ± 75.47 min, 44.73 ± 12.17 min, and 38.52 ± 25.78 min, respectively. During the procedure, minor LAAO recaptures occurred in 45.45 % of cases, and complete recaptures occurred in 13.64 % of cases. None of the study patients had a leak on the device > 3 mm by TEE, LAAO implant dislodgement, cardiac tamponade, or major cardiovascular events, including cardiovascular death, myocardial infarction, ischemic stroke, or transient ischemic attack during the periprocedural period.

We then compared the performance of 3D CT, TEE, and intra-procedural LA angiography in determining the LAA ostium diameter against the actual device size and the optimal compression ratio attainment rate. The median size of the LAA ostium measured by central line-based 3D CT (24.3 mm, IQR = 22.0–27.0 mm) was closer to the actual size of the Watchman device (27.0 mm, IQR = 24.0–31.0 mm, P = 0.127) in comparison with that assessed by TEE (21.2 mm, IQR = 18.4.0–22.7 mm, P < 0.001) and LA angiography (22.5 mm, IQR = 17.9–25.1 mm, P < 0.001).

**Fig. 4:**
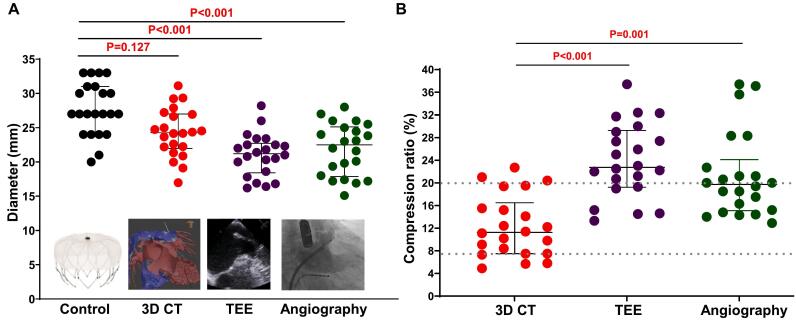
**Comparison of different imaging modalities.** (A) Comparison of different imaging modalities with the actual size of the implanted occluder. The analysis shows that the LAA ostium diameter (perimeter-derived diameter) obtained using the 3D CT algorithm is more accurate and closer to the actual occluder size than the diameters obtained by TEE or angiography. (B) Compression rates were determined by various imaging modalities, highlighting that 3D CT provides a more precise compression rate. Abbreviations: 3D CT, 3D Computed Tomography; TEE, transesophageal echocardiography.

The compression ratio was calculated as follows: (actual implantation size − predicted size) / actual implantation size. Preprocedural 3D CT measurement was associated with a better attainment rate in achieving the optimal median compression ratio than TEE (10.8 %, IQR = 7.4–16.5 % vs. 22.7 %, IQR = 19.2–29.3 %, P < 0.001) and LA angiography (19.7 %, IQR = 15.1–24.1 %, P = 0.001) (Fig. 4).

During a median follow-up period of 609 days (IQR, 513.5–675.5 days), none of the study patients had any leak on the device by TEE, LAAO implant dislodgement, stroke, or heart failure rehospitalization, with 36.36 % of patients receiving antiplatelet therapy, 54.55 % on anticoagulants, 4.55 % on warfarin, and 4.55 % on no medication. No all-cause mortality was observed (Table 2).

**Table 2:** Procedural and clinical outcomes.

**Variables**	**N = 22**
**Procedure duration**	
One-stage procedural time (min)	165.59 ± 75.47
LAAO implant time (min)	44.73 ± 12.17
Total fluoroscopy time (min)	38.52 ± 25.78
**Pulmonary veins isolation type**	
Radiofrequency catheter ablation	18 (81.82 %)
Cryoballoon ablation	4 (18.18 %)
**Pulmonary veins isolation success rate**	22 (100 %)
**Device type**	
Watchman	18 (81.82 %)
Watchman FLX	4 (18.18 %)
**Size of the final device**	
Watchman	27.0 (24.0–30.0)
Watchman FLX	29.0 (21.8–31.0)
**Contrast volume (mL)**	105.0 (81.5–150.0)
**Reposition of LAAO**	
Minor recapture	10 (45.45 %)
Fully recapture	3 (13.64 %)
**Periprocedural outcomes**	
Leak on the device > 3 mm	0 (0 %)
LAAO dislodgement	0 (0 %)
Cardiac tamponade	0 (0 %)
Stroke or TIA	0 (0 %)
Major cardiovascular events	0 (0 %)
**Antithrombotic treatment at discharge**	
Antiplatelet agents	8 (36.36 %)
Non-vitamin K antagonist oral anticoagulants	12 (54.55 %)
Warfarin	1 (4.55 %)
None	1 (4.55 %)
**Follow-up (days)**	609.0 (513.5–675.5)
All-cause mortality	0 (0 %)
Stroke	0 (0 %)
Heart failure rehospitalization	0 (0 %)
Any leak on the device	0 (0 %)
LAAO dislodgement	0 (0 %)
Recurrent atrial tachyarrhythmias	5 (25 %)

Data are presented as mean ± SD or median (IQR) and n (%). LAAO, left atrial appendage occlusion; major cardiovascular events include cardiovascular death, myocardial infarction, and ischemic stroke.

## 4 Discussion

This is the first report demonstrating that interactive, central line-based 3D CT planning enables the selection of a more appropriate implant device size than measurements obtained using TEE or LAA angiography in one-stage PVi with a concomitant LAAO procedure [10], [11], [12], [13]. Unlike TEE and angiography, 3D CT planning provides a more comprehensive anatomical overview, potentially reducing sizing discrepancies and enhancing procedural planning by integrating detailed spatial information about the LAA and its surrounding structures in a combined procedure.

### 4.1 One-stage AF ablation with concomitant LAAO

The “one-stage procedure” for AF is crucial, as it provides comprehensive stroke prevention and symptom improvement while exposing patients to procedural risks only once. Combining LAAO with PVi in a one-stage procedure enhances stroke prevention and may reduce or eliminate the need for anticoagulants [2], [14]. Data from international registries indicate a significant reduction in the annualized stroke rate compared to expected values based on CHA_2_DS_2_-VASc scores in patients undergoing the combined procedure [15], [16]. It has been shown that in symptomatic patients with AF with high stroke and bleeding risk who are planned for AF ablation, the combined AF ablation and LAAO procedure may be a cost-effective therapeutic option and be more beneficial to patients with CHA_2_DS_2_-VASc risk score ≥ 3 [17].

In the one-stage procedure, it is important to determine the sequence of LAAO and catheter ablation for the PVi and to consider the impact of different transseptal puncture sites. Typically, to minimize the impact of the LAAO device on the left-sided PVi and reduce the risk of device dislodgement, most teams, including ours, first perform PVi, followed by LAAO. However, performing ablation can significantly alter the left atrial LAA ostium shape because of induced edema, thereby complicating the intraoperative TEE assessment of the ostium size [2]. Compared to RF ablation, cryoballoon ablation is more likely to alter the shape of the LAA ostium, potentially interfering with LAAO to a greater extent. To date, the impact of these two techniques on subsequent LAAO procedures remains unclear. Consequently, relying on intraoperative TEE guidance to ascertain whether the LAAO device is adequately sealed or stable can result in a significant misjudgment. Therefore, a precise preoperative assessment of the shape and size of the LAA ostium is imperative for the success of the procedure.

### 4.2 Innovative preprocedural 3D CT planning

We developed a preprocedural interactive central line-based 3D CT navigation system to accurately assess the appropriate device size for successful implantation in one-stage PVi with concomitant LAAO procedures. This system efficiently helps operators understand the relationship between the LAA landing zone and adjacent structures. It automatically distinguishes the atrial chamber spaces using a region-growing method based on the HU thresholds in the original 3D image. The system calculates the central axis from the oval fossa puncture point to the LAA lobe, typically the anterior lobe, using continuous inscribed circles to determine a suitable LAA ostium position. The diameter, perimeter, and cross-sectional area of the LAA ostium were calculated.

For the ostium diameter calculation, the perimeter-derived diameter method, similar to that used for self-expanding valves in transcatheter aortic valve replacement, was employed. This method accurately represents the size after the occluder, and the LAA endocardium achieves balanced adhesion. Despite the irregular circumference of the LAA ostium, our system accurately determined the LAA boundary using an algorithm-based process and obtained the perimeter-derived diameters at various implantation depths. This preprocedural planning yielded an LAA ostium diameter closer to the actual size of the implanted occluder, and an appropriate compression rate. In contrast, the LAA ostium diameters measured by TEE and LAA angiography are often underestimated, increasing the risk of device dislodgement or peri-device leakage in one-stage procedures. This study also demonstrated that the interventionist selected device size, based on the measurements obtained from TEE and LAA angiography combined with personal experience, aligns more closely with the results derived from the interactive central line-based 3D CT planning method. This finding suggests that an automated CT algorithm can provide more objective and accurate data, thus reducing the reliance on an interventionist's personal experience. Consequently, it may mitigate the risk of selecting an oversized device in cases with insufficient experience, lowering the likelihood of periprocedural complications, such as LAA rupture.

### 4.3 Procedural and clinical outcomes

Previously, LAAO device strategies relied heavily on the interventionists' experience to achieve optimal compression rates and implant depths, especially in one-stage procedures, where tissue edema post-ablation affects device stability and may cause residual leakage. A higher compression ratio achieved using a larger device risks LAA rupture because of excessive expansion, or may cause the device's extension height to exceed the available LAA depth, leading to shoulder protrusion and device instability or dislodgement [18], [19]. It is important to note that myocardial tissue edema following ablation may cause the intraoperative compression rate to appear higher than normal. However, once the tissue edema subsides, the compression rate may become insufficient, potentially resulting in device instability or peri-device leakage. Thorough preoperative imaging assessment methods should be used to ensure the success in a one-stage procedure.

In this proof-of-concept study, using an innovative preprocedural interactive central line-based 3D CT planning for one-stage PVi with LAAO, we achieved a 100 % procedural success rate among 22 patients, with no perioperative complications, such as device dislodgement, cardiac tamponade, residual leakage >3 mm, or periprocedural stroke. During the postoperative follow-up, there were no incidents of death, stroke, heart failure, or hospitalization.

Newer generation LAAO devices, such as the Watchman FLX, are more flexible and conformable to LAA tissue, reducing the emphasis on compression ratios [20]. However, understanding the appropriate depth and diameter of the LAA opening after achieving a near-circular shape with the occluder remains crucial for interventionists when selecting device size and planning procedural steps preoperatively.

## 5 Limitations

In this proof-of-concept, small-scale pilot study, we demonstrated the usefulness of an innovative preprocedural interactive central line-based 3D CT planning approach in guiding one-stage AF ablation with concomitant LAAO. Our study had several limitations. First, this retrospective, nonrandomized study included a relatively small sample size of 22 consecutive patients. This limited sample size may not adequately represent the diverse spectrum of patients with AF undergoing PVi with concomitant LAAO. The findings derived from this small cohort may not be generalizable to larger populations with varying clinical characteristics, comorbidities, or anatomical variations. Second, this study exclusively utilized the Watchman device, excluding the Amulet device, which requires less consideration of the compression rate. Consequently, these findings may only be applicable to procedures involving Watchman devices. Third, the study was conducted at a single center, potentially introducing biases related to patient selection, procedural techniques, and institutional practices. The outcomes observed in this study may have been influenced by center-specific factors, such as institutional expertise, available equipment (e.g., the absence of an ICE at our institution during the study period), and local protocols. These variations may limit the generalizability of the results to other centers with different resources and clinical practices. Fourth, although the study metrics provide valuable insights into procedural planning, device sizing, and mid-term clinical outcomes such as stroke risk reduction, peri-device leak, and thromboembolic events, long-term clinical outcome assessment is needed to validate the usefulness of using preprocedural interactive central line-based 3D CT planning in guiding one-stage PVi with a concomitant LAAO procedure. Fifth, to justify the additional burden of CT scans, including exposure to radiation and contrast media, it is essential to demonstrate the potential clinical benefits in adequately powered trials. This consideration is crucial, given the notable fluoroscopy duration associated with the procedure in this population. Finally, the comparison focused solely on the performance of interactive central line-based 3D CT planning, TEE, and intraoperative LA angiography in assessing the LAA ostium diameter and guiding device sizing. Other imaging modalities, such as cardiac magnetic resonance imaging or ICE, were not evaluated. Therefore, the conclusions of this study may not encompass the full spectrum of available imaging modalities for the preprocedural planning of LAAO procedures. Furthermore, in real-world practice, the choice of device size should be based on measurements obtained from preprocedural CT scans, TEE, LAA angiography, and possibly ICE. Further studies are required to address these limitations through larger-scale multicenter studies with longer follow-up durations, inclusion of diverse patient populations, comprehensive assessment of clinical outcomes, evaluation of additional imaging modalities, and implementation of randomized procedures to enhance the robustness and generalizability of the findings in guiding optimal device sizing and selection in patients with AF undergoing PVi with concomitant LAAO.

## 6 Conclusion

The pilot study results suggest that using preprocedural interactive central line-based 3D CT planning to assess the LAA ostium diameter to guide device size selection may be more effective than intraoperative TEE or left atrial angiography in patients with AF undergoing a combined PVi and LAAO procedure with the Watchman device. Further large-scale studies are necessary to confirm the generalizability of this finding and to guide the optimal device sizing and selection in patients with AF undergoing PVi with concomitant LAAO.

## CRediT authorship contribution statement

**Ke-Wei Chen:** Writing – review & editing, Writing – original draft, Validation, Supervision, Methodology, Investigation, Funding acquisition, Data curation. **Yen-Nien Lin:** . **Mei-Yao Wu:** . **Yi-Hsiu Wu:** Methodology, Investigation, Data curation. **Wen-Sheng Feng:** Supervision, Methodology. **Ping-Han Lo:** Investigation. **Wei-Hsin Chung:** Investigation. **Cheng-Chang Tung:** Investigation. **Kuan-Cheng Chang:** Writing – review & editing, Writing – original draft, Validation, Supervision, Methodology, Investigation, Funding acquisition, Data curation.

## Funding

This study was supported in part by the Taiwan Ministry of Science and Technology [NSTC 112-2314-B-039-031, MOST 111-2314-B-039-012, and MOST 110-2314-B-039-050] and China Medical University Hospital [C1110812016-8, C1110831002-11, DMR-111-020, and DMR-110-012]. None of the funding sources played a role in the study design, data collection, analysis, interpretation, report writing, or decision to submit the paper for publication.

## Declaration of competing interest

The authors declare that they have no known competing financial interests or personal relationships that could have appeared to influence the work reported in this paper.
